# Anticoagulant interruption in transcatheter aortic valve implantation (TAVI): a strategy to lower bleeding risks

**DOI:** 10.1097/MS9.0000000000003285

**Published:** 2025-04-10

**Authors:** Sana Rasheed, Muhammad Anas, Izere Salomon

**Affiliations:** a Department of Medicine, Jinnah Sindh Medical University, Karachi, Pakistan; b Bacha Khan College of Dentistry Mardan, Pakistan; c Department of General Medicine and Surgery, University of Rwanda College of Medicine and Health Sciences, Kigali, Rwanda

*Dear Editor*,

Transcatheter Aortic Valve Implantation (TAVI) has emerged as a new therapeutic modality for aortic stenosis, especially in high-risk patients[1]. Despite the reliability and advantages of this process, it has some drawbacks, including the issue of bleeding resulting from anticoagulation therapy. Strategically interrupting anticoagulants may help reduce these risks, leading to better patient outcomes[1]. Managing coagulation in TAVI is complex as anticoagulants are routinely prescribed to prevent thromboembolic complications[1]. About one-third of patients undergoing TAVI for severe aortic stenosis require long-term anticoagulation therapy. This typically involves either vitamin K antagonists (VKAs) or direct-acting oral anticoagulants (DOACs)^[^2,3^]^. In clinical practice, anticoagulation is typically paused 2-4 days before TAVI[4]. The approach to anticoagulation in TAVI may differ from percutaneous coronary intervention (PCI) due to differences in patient population, disease, and implanted device[5]. Combining DAPT with anticoagulants increases bleeding risk, especially in patients requiring long-term oral anticoagulation[5]. However, research suggests that using these medications after the procedure may increase the risk of bleeding complications, particularly when used around the time of TAVI[6]. Therefore, strategically interrupting anticoagulation before the procedure may help minimize bleeding risks while still protecting against thrombosis. Anticoagulation therapy is essential in TAVI due to the high risk of thromboembolic and bleeding complications[7]. Current TAVI anticoagulation standards recommend using unfractionated heparin to achieve an ACT above 300 seconds, followed by reversal with protamine sulfate after the procedure. 3 Post-TAVI patients are prescribed dual antiplatelet therapy with aspirin and clopidogrel. Aspirin is continued indefinitely, while clopidogrel is prescribed for 3 to 6 months, though recent debates question the optimal duration and necessity of this regimen[3]. Bleeding complications from these medications can be severe, even life-threatening. Studies have shown that 15-32% of patients experience bleeding complications, with underlying conditions like anemia further increasing the risk[7]^.^ These bleeding risks emphasize that anticoagulant therapy has to be approached cautiously to prevent new thromboembolic events but at the same time to minimize the risk of major bleeding[7].

**HIGHLIGHTS**

- Strategically interrupting anticoagulant therapy during transcatheter aortic valve implantation (TAVI) is associated with a significant reduction in bleeding complications.
- Individualized perioperative management plays a crucial role in minimizing thromboembolic risks while ensuring patient safety.
- Findings from the BRAVO-3 trial provide support for the interruption of anticoagulant therapy without compromising patient outcomes.
- Current guidelines emphasize the implementation of tailored anticoagulation strategies based on patient-specific risk profiles.
- Future large-scale trials are imperative to further optimize anticoagulation protocols in the context of TAVI.

A recent Cochrane systematic review examined the effects of temporarily discontinuing VKA or DOAC therapy and found it reduces the risk of major and life-threatening bleeding. Several studies have shown that patients experiencing TAVI with anticoagulant interruption had fewer complications; hence, there is a need to improve patient safety by effectively managing anticoagulation^[^7,8^].^ This is particularly important when identifying appropriate candidates for anticoagulant therapy interruption and implementing it in clinical practice. Factors such as a patient’s frailty, overall morbidity, and the specific procedural risk profile should all be carefully considered. For instance, patients who have been presented with bleeding events, or those who are likely to develop thromboembolic events, need to be considered for a working treatment[8]. In summary, the approach should involve systematic and individualized evaluations of patients to optimize outcomes after TAVI while minimizing bleeding^[^4,5^]^.

Pausing anticoagulants during TAVI introduces potential thromboembolic risks, such as stroke and valve thrombosis, particularly in patients with conditions like atrial fibrillation or mechanical heart valves. The interruption of anticoagulation increases the risk of clot formation, which must be carefully balanced against the bleeding risks associated with continued anticoagulation during the procedure^[^8,9^]^.

Counterarguments emphasize the potential dangers of thromboembolism, particularly in high-risk patients, where even brief interruptions could lead to adverse outcomes^[^10,11^]^. However, these risks can be mitigated through strategies such as bridging anticoagulation with short-acting agents like low-molecular-weight heparin and vigilant perioperative monitoring^[^10,11^]^. The study suggests that the timely resumption of anticoagulants post-TAVI, combined with individualized risk assessment, can successfully balance the reduction in bleeding risks with manageable thromboembolic risks[6]. Additionally, the safety of this approach indicates that when the anticoagulant interruption is carefully implemented, it can achieve a favorable balance between preventing thromboembolic events and minimizing bleeding complications^[^10,11^]^.

Implementing anticoagulant interruption during TAVI requires strict adherence to clinical guidelines and individualized patient assessments. The 2023 ESC/EACTS guidelines emphasize evaluating each patient’s thromboembolic and bleeding risks to tailor anticoagulation management. For example, in a case study, an 82-year-old male with severe aortic stenosis and atrial fibrillation underwent TAVI with anticoagulation interrupted and bridged using enoxaparin. The patient experienced no significant bleeding or thromboembolic events, demonstrating the effectiveness of careful anticoagulation management[12].

Similarly, recent high-quality studies and meta-analyses provide critical insights into optimizing antithrombotic strategies. A study involving 668 patients undergoing TAVI demonstrated that the continuation of oral anticoagulation led to fewer thromboembolic events without an increase in major bleeding, supporting the continuation of oral anticoagulant as a safe strategy for selected patients[13]. A network meta-analysis further highlights the importance of individualized approaches, analyzing 12 studies and finding that combination antithrombotic therapy, such as dual antiplatelet therapy, effectively reduced thromboembolic risks but was associated with increased bleeding rates[14]. This highlights the need for careful patient selection when considering more aggressive antithrombotic regimens. Similarly, another study reviewed 14 studies and concluded that dual antithrombotic strategies provide the optimal balance between thromboembolic prevention and bleeding risk, particularly in high-risk populations[15]. Their findings emphasize the importance of tailoring therapy based on individual patient profiles.

Monitoring during the perioperative period is essential, with close observation of coagulation parameters and signs of bleeding or thromboembolism. Immediate postoperative follow-up, particularly within the first 30 days, is crucial for early detection of any complications[10]. These examples underscore the importance of individualized protocols in safely implementing anticoagulant interruption in TAVI patients, balancing the risks while optimizing outcomes^[^10,11^]^.

In conclusion, the management of anticoagulation in TAVI remains a dynamic and evolving area, requiring a balance between thromboembolic prevention and bleeding risk. This editorial synthesizes emerging evidence and highlights the need for individualized strategies over a one-size-fits-all approach. By integrating recent meta-analyses and guidelines, we propose a nuanced framework for anticoagulant interruption, emphasizing patient-specific risk stratification. Future research should refine predictive models, explore safer antithrombotic agents, and evaluate real-world outcomes. Advancing this field will require a multidisciplinary effort to establish best practices and improve patient safety.

## Data Availability

Not applicable.
